# Artificial Faces Predict Gaze Allocation in Complex Dynamic Scenes

**DOI:** 10.3389/fpsyg.2019.02877

**Published:** 2019-12-18

**Authors:** Lara Rösler, Marius Rubo, Matthias Gamer

**Affiliations:** Department of Psychology, Julius-Maximilians-Universität Würzburg, Würzburg, Germany

**Keywords:** social attention, faces, physical saliency, visual perception, naturalistic scenes, eye movements

## Abstract

Both low-level physical saliency and social information, as presented by human heads or bodies, are known to drive gaze behavior in free-viewing tasks. Researchers have previously made use of a great variety of face stimuli, ranging from photographs of real humans to schematic faces, frequently without systematically differentiating between the two. In the current study, we used a Generalized Linear Mixed Model (GLMM) approach to investigate to what extent schematic artificial faces can predict gaze when they are presented alone or in competition with real human faces. Relative differences in predictive power became apparent, while GLMMs suggest substantial effects for real and artificial faces in all conditions. Artificial faces were accordingly less predictive than real human faces but still contributed significantly to gaze allocation. These results help to further our understanding of how social information guides gaze in complex naturalistic scenes.

## Introduction

When exploring our surroundings, we preferentially allocate attention to other human beings. Various eye-tracking studies have shown that our strong tendency to fixate others is apparent both when viewing images or videos in laboratory settings (Itier et al., 2007; Birmingham and Kingstone, 2009; Cerf et al., 2009; Kingstone, 2009; Bindemann et al., 2010; Coutrot and Guyader, 2014; Xu et al., 2014; Nasiopoulos et al., 2015; End and Gamer, 2017; Flechsenhar and Gamer, 2017; Rösler et al., 2017) and, although to a slightly reduced extent, in real-life social interactions (Foulsham et al., 2011; Laidlaw et al., 2011; Freeth et al., 2013). Among these different viewing modalities, a strong preference for heads (Freeth et al., 2013) and, if stimulus resolution allows, eyes of others (Birmingham et al., 2008) can be discerned. It has been argued that this bias toward the eyes of conspecifics enables the deciphering of others’ internal states and therefore represents an essential prerequisite for successful social interactions and integration in society (Shimojo et al., 2003; Ristic et al., 2005; Frischen et al., 2007).

We are sometimes, however, confronted with human-like features which do not give room for interaction. We here refer to any human-like face that has been produced by another human being as artificial. By this definition, an advertisement poster of a local politician but also a statue in a church or a humanoid robot are considered instances of artificial faces. How does the processing of these artificial faces differ from the processing of real faces? Mimicry and gesture of cartoon figures or statues also convey information about their alleged emotions or internal states and were even seen to yield higher accuracies in emotion detection than real faces (Kendall et al., 2016). Observers yet commonly know that these human representations are not real and therefore cannot be meaningfully interacted with. Previous studies have shown that gaze patterns are affected by social presence (Freeth et al., 2013) and the possibility of a social interaction (Laidlaw et al., 2011), leading to gaze behavior that is adapted to social norms (e.g., reduced fixations on strangers’ heads). The attentional bias toward eyes was yet seen to persist even when these are part of very unhuman-like fictional monsters and located in surprising parts of their bodies (Levy et al., 2013). The similarity between artificial and real human face processing is further highlighted by a vast body of electrophysiological studies which reported neural face-processing signatures, e.g., the electrophysiological N170 response in the electroencephalogram, to schematic faces (Jeffreys, 1996), inverted schematic faces (Sagiv and Bentin, 2001) and even scrambled face features after face priming (Bentin and Golland, 2002; Bentin et al., 2002). However, effects of direct vs. averted gaze in these electrophysiological responses could only be detected with photographic but not with schematic faces (Rossi et al., 2015) and overall the amplitude of the N170 largely seems contingent on the fixation of eyes (Itier et al., 2007; Parkington and Itier, 2018). Functional magnetic resonance imaging studies have further shown that similar brain regions are recruited when perceiving a performed action (Gazzola et al., 2007) or emotion of a robotic or human agent (Chaminade et al., 2010). Interestingly, a preference of face-like artificial stimuli could even be observed in the human fetus (Reid et al., 2017; but see Scheel et al., 2018), yielding initial evidence that our tendency to orient to artificial face-like structures is not contingent on postnatal experience. These findings suggest that social features attract attention even when they are not part of an actual fellow human being. How are fixations distributed, however, when both real and artificial faces directly compete for attentional resources?

For the further exploration of processing differences between real and artificial faces the choice of appropriate stimulus material is a challenging one. While static images can in theory display both artificial and real human faces, they will ultimately be an instance of artificial material (e.g., a picture of a person viewing a picture of a person). Videos, however, allow the possibility to display both real human and artificial faces while rendering the difference between the two more evident. Furthermore, videos are a better approximation of real-life dynamic situations than static stimuli potentially rendering the interpretation of results more meaningful (Risko et al., 2012). Accordingly, computational accounts of gaze allocation perform significantly better when motion, which is only available during dynamic and not static stimuli, is considered during face processing analyses (Curio et al., 2011). The superiority of dynamic stimuli in face processing research is further supported by clinical studies showing that certain differences in gaze allocation between patients with autism spectrum disorder and healthy controls only become apparent when using dynamic instead of static stimuli (Speer et al., 2007).

There is an on-going scientific debate to what extent low-level physical features of the stimulus material (so-called physical saliency) need to be considered when analyzing gaze patterns in static or dynamic scenes. While proponents of saliency approaches claim that bottom-up processing of scenes can be fully accounted for by low-level physical features such as luminance, color intensity and orientation (e.g., Itti et al., 1998; Itti and Koch, 2000), various studies have shown that these algorithms do not work well when top-down influences are strong (as reviewed by Tatler et al., 2011). The use of dynamic stimuli, however, introduces additional temporal saliency features (e.g., flicker and motion) which were seen to predict viewing behavior during free-viewing (Mital et al., 2011) supporting the general notion of low-level physical saliency as a crucial predictor of gaze allocation.

To disentangle the influences of physical saliency and the appearance of human and artificial faces on gaze patterns, we presented videos including human faces only, artificial faces only and videos including both human and artificial faces to participants while recording their eye movements. Saliency maps were computed using the Graph Based Visual Saliency (GVBS) algorithm first introduced by Harel, Koch, and Perona (Harel et al., 2007). Using a generalized linear mixed model (GLMM), we were able to separately evaluate the impact physical saliency and human and artificial faces had on fixation probability. Since participants freely viewed the stimulus material, we expected human faces and low-level physical saliency to be most impactful on eye movements but assumed artificial faces to also attract attention although to a somewhat lesser extent.

## Materials and Methods

### Participants

A prior power analysis (Faul et al., 2007) showed that 34 participants were necessary for revealing medium-sized effects in paired *t*-tests (Cohen’s *d* = 0.50) at a significance level of α = 0.05 and a power of 0.80. In order to take into account potential dropouts, we recruited thirty-six participants (15 males). Because of a too large variability of baseline coordinates (for calculations see below), one participant had to be excluded from our sample. Our final sample thus consisted of 35 participants with a mean age of 25.66 years (*SD* = 4.88 years) via the University of Würzburg’s Human Participant Pool. All participants had normal or corrected-to-normal vision. Ethical approval was obtained by the Ethics Committee of German Psychological Society (DGPs). Each participant provided written informed consent and was awarded monetary compensation or course credit for participation.

### Stimuli

The stimulus set consisted of a total of 60 videos varying between 18 and 20 s of length without any cut interruption. These 60 videos contained four subsets of 15 videos each displaying either only real human faces, only artificial faces, both human and artificial faces, or no faces at all. Artificial faces were categorized as such when they shared key features of a human face including round shape, nose and eyes but did not belong to an actual human being in the scene. Examples include posters of humans, statues or street art (for a detailed description of the video content see Supplementary Table 1). The 30 videos including artificial faces were newly acquired via a free online streaming platform, while the remaining 30 videos were taken from an earlier study (Rubo and Gamer, 2018). In order to be included in our study, videos generally had to depict natural scenes, usually representing outdoor scenery, and had to be taken from a wide angle with a still or slowly moving camera. Additionally, the human beings displayed in the videos were not to perform any surprising actions. As text is known to greatly influence gaze allocation (Cerf et al., 2009), we further attempted to avoid the display of conspicuous text within our videos. All videos had a resolution of 1280 × 720 pixels and were converted from their original format to a 30 frame-per-second MPEG-4 video file resulting in a total of 35,041 frames across all videos.

### Apparatus

Videos were presented centrally on 24′′ LG 24MB 65PY-B screen (516.9 × 323.1 mm; 1920 × 1200 pixels, 60 Hz). We used a chin and forehead rest to minimize head movements and to warrant a constant viewing distance of 50 cm, resulting in a viewing angle of 38.03°× 21.94 of the displayed videos. Eye movements of the right eye were tracked at a sampling rate of 1,000 Hz (EyeLink 1000 Plus, SR Research, Oakville, ON, Canada). Stimuli were presented using MATLAB© 2011b (Mathworks, Inc., Natick, MA, United States) and the Psychophysics Toolbox (Version 3.0.12) (Brainard, 1997; Pelli, 1997; Kleiner et al., 2007).

### Procedure

Prior to data acquisition, participants were instructed to watch the videos as if watching a TV-show. To avoid fatigue, the experiment was split into two blocks, each containing 30 videos. Each trial began with a fixation cross displayed centrally on a gray background for 5–9 s, followed by the onset of a video. Eye movements were recorded together with time stamps marking the beginning of each video frame. To avoid sequence effects, videos were displayed in random order to each participant. As a final part of the experiment, participants filled in various psychometric tests and questionnaires which will be pooled across several studies and are not analyzed as part of this manuscript.

### Eye Tracking Preprocessing

Gaze data were analyzed using R (version 3.2; R Development Core Team, 2015). Any eye tracking data recorded up until 150 ms after stimulus onset were excluded from the analysis to account for lingering on the initial fixation cross position. Since the eye tracker sampled eye movements at 1,000 Hz and videos had a frame rate of 30 Hz, approximately 33 raw eye positions were recorded per frame. Eye data was consequently collapsed over each frame such that fixation coordinates refer to the mean of these 33 raw eye positions per frame. Baseline *x* and *y* coordinates were calculated as the mean fixation positions 300 ms before stimulus onset. Similar to our previous studies (e.g., End and Gamer, 2017; Rubo and Gamer, 2018), baseline outliers were identified by an iterative outlier removal procedure which was conducted separately for *x*- and *y*-coordinates. Specifically, the largest and smallest values were removed temporarily from the distribution. If any of these extreme values was more than three standard deviations from the mean of the remaining distribution, it was permanently excluded. Otherwise, the values were returned to the distribution. This procedure was then repeated until no more exclusions had to be performed. Subsequently, missing baselines (*M* = 9.55% of all trials across participants, *SD* = 9.16%) were replaced by the mean baseline of all valid trials and, to account for gaze drifts, baseline coordinates were then subtracted from the gaze data of each trial. Frames were excluded from analyses if the corneal reflection was lost during blinks or large eccentricity fixations and if gaze was directed toward a position outside of the video area (*M* = 2.12% of all data points for each participant, *SD* = 3.05%).

### Influence of Saliency, Region of Interests and Distance to Center

To investigate the influence of physical saliency on gaze allocation, we calculated saliency maps for each frame of each video. These maps were created using the GVBS algorithm (Harel et al., 2007) which takes luminance, color, orientation and flicker with equal weights into account and has been shown to have high prediction accuracy (Judd et al., 2012). We additionally applied Gaussian blurring along the temporal dimension of the video data to reduce the influence of strong changes in low-level saliency between successive video frames (Rubo and Gamer, 2018). These saliency values were normalized to have a mean of 1. Regions of interests (ROIs) for human and artificial faces were defined manually using circular masks. Video locations that included a face were coded as 1 whereas the remainder of the frame was coded as 0. Finally, we modeled a predictor for center bias by calculating the inverse Euclidean distance of scene locations to the center of the video.

In order to estimate the relative contribution of these predictors on gaze allocation, we aggregated data across 40 × 40 pixels patches that were arranged in a regular 32 × 18 grid. This grid size was already used in a previous study (Rubo and Gamer, 2018) and approximates the size of the functional field of the human fovea centralis at the current viewing distance. For each feature map (i.e., physical saliency, human and artificial faces, centrality), we calculated mean values for each of the 576 cells of the grid. Finally, values were *z*-standardized across each map to allow for comparison of the beta coefficients in the statistical analyses.

### Statistical Analyses

As a first analysis, we calculated fixation durations per ROI (human and artificial faces) weighed by ROI size and ROI presentation duration per video category. To this end, we summed the number of frames per video in which the looked-at grid cell contained a human or artificial face separately for each ROI per video category and divided it by the number of pixels the ROI made up within the cell in each iteration. The resulting fixation count, corrected for ROI size, was then divided by the number of frames which contained that ROI type per video. As the average fixation durations per participant were not normally distributed, we subsequently submitted these values to two Wilcoxon signed rank tests, one contrasting human face fixations in the human video category with artificial face fixations and another contrasting the fixations of human and artificial faces in the videos in which both faces are presented simultaneously.

We furthermore determined fixation latencies as the point in time when each ROI was first fixated in each video by each participant. These values were aggregated individually for each participant, across all videos of the same type (i.e., videos containing only real faces vs. only artificial faces vs. both real and artificial faces). Some participants never looked at a ROI in some of the videos. On average, this was the case for 0.51 (*SD* = 0.95, range = 0–4) videos containing only real human faces and 0.14 (*SD* = 0.55, range = 0–3) videos containing only artificial faces. In the videos containing both real and artificial faces, no real face was looked at in 0.17 (*SD* = 0.71, range = 0–4) of the videos, and no artificial face was looked at in 1.17 (*SD* = 1.32, range = 0–7) of the videos. Analyses therefore focused on the subset of videos within each participant in which a specific ROI was regarded at least once. Since latencies were not normally distributed, we again performed Wilcoxon signed rank test to first compare latencies for real and artificial face fixations in the videos containing only one face type and subsequently in the videos in which both faces were presented simultaneously. Effect sizes for all Wilcoxon signed rank test were calculated according to the suggestion of Rosenthal (1994) with the formula *r* = *Z*/√*N*.

**FIGURE 1:**
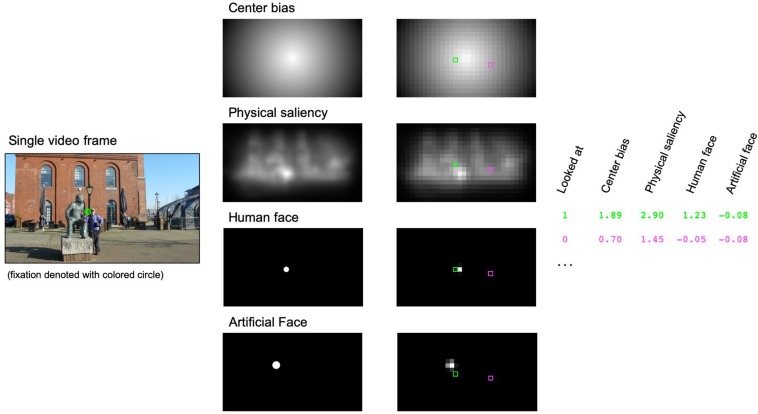
Illustration of the generalized linear mixed model (GLMM) approach for predicting individual fixations. A sample video frame with a possible fixation is shown on the left side. For each video frame, we defined center bias and physical saliency [as calculated by the Graph Based Visual Saliency (GVBS) algorithm] and circular regions of interest for human and/or artificial faces. These maps (middle column) were tiled into a regular 32 × 18 grid with individual cells reflecting the average of the raw values within each cell (right column). Finally, values were *z*-standardized within each map. Within the GLMM approach, we tried to predict whether a given cell was looked at (here denoted with a green square) or not (randomly selected cell marked with a magenta square) by using center bias, physical saliency and, if appropriate, the presence of human and/or artificial faces. Please note that the image depicted here was not part of the videos and is only shown for illustration.

To more elaborately investigate the individual contributions of centrality, physical saliency, human and artificial faces on gaze allocation, we calculated nine separate GLMMs in R using the package lme4 (Bates et al., 2014) and the *bobyqa* optimizer. Mixed-effect models have been explicitly suggested as an excellent tool to predict fixation patterns in naturalistic scenes based on image features (Nuthmann and Einhäuser, 2015). The criterion variable in these models was defined by the current fixation in each video frame. In order to reduce biases between looked at and not-looked-at locations in the statistical analyses, two cells of the 32 × 18 grid were selected for each video frame and used in the GLMM. This included the currently fixated cell, as revealed by the eye-tracking data, and one randomly chosen non-fixated cell. The response variable thus described whether a grid cell was fixated or not and we chose to model this binary event using a binomial error distribution and the probit link function. Centrality, physical saliency, human and artificial faces served as quantitative predictors in the models (see Figure 1 for an illustration of the procedure).

We used an incremental approach and initially calculated a simplified model which only included distance to center and saliency values as fixed predictors for each video category. Secondly, we added the respective ROI predictors (i.e., human and artificial faces) in a separate model for each video category, yielding a total of two models for both the real human faces and the artificial face videos. For the videos containing both artificial and human faces our incremental approach yielded four different models, the simplest one including only saliency and distance to center as predictors, one model adding only one of the respective ROIs and a final model including both ROIs in addition to the saliency and centrality predictors. To account for within-subject and within-video effects, subject and video numbers were entered as random intercepts. We considered the size of beta weights (β) to estimate which predictor predominantly influenced gaze allocation and evaluated *R*^2^ of the models to assess which model performed best. As the non-fixated grid cell was randomly chosen for each frame of each video, we decided to apply a bootstrapping procedure to validate our model outcomes and to ensure that results do not depend on an individual selection of cells. Herein, the process of randomly choosing a non-fixated grid cell was repeated over 100 iterations and 100 respective GLMMs were calculated for each of the nine different models. Based on the results of this bootstrapping procedure, we subsequently calculated mean beta weights, mean *R*^2^ and 95% Confidence Intervals (CIs) for each predictor and considered beta weights and *R*^2^ significantly different from one another when the CIs did not overlap.

## Results

### Fixation Durations

**FIGURE 2:**
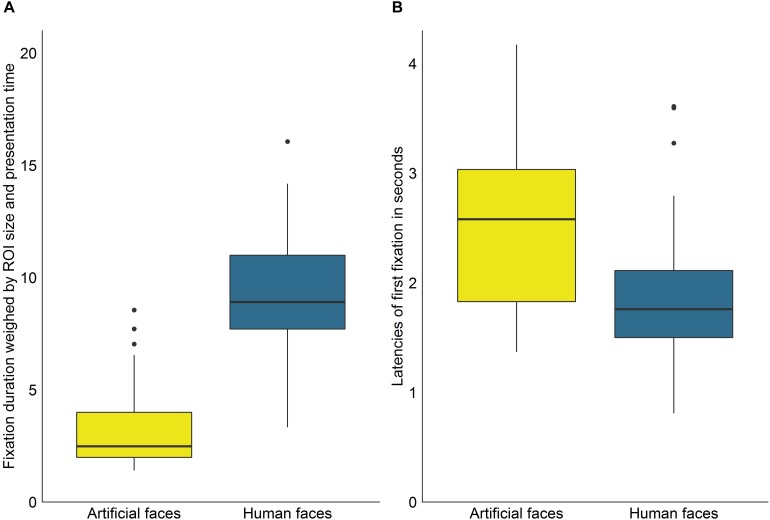
**(A)** Average duration of fixations on artificial and human faces weighed by ROI size per frame and presentation time of ROIs per video. **(B)** Average latencies of fixations on artificial and human faces. Outliers are defined as points further than 1.5 ^∗^ interquartile range of the lower or upper hinge.

To investigate whether fixation durations differed significantly between ROIs, we first calculated a Wilcoxon signed rank test comparing fixations on human faces in the videos in which exclusively human faces were shown with fixations on artificial faces in the videos in which only the artificial faces were shown. The results revealed no significant differences between the two face types (*W* = 717, *p* = 0.089, *r* = 0.23). As the comparison rests on two entirely different sets of videos, we subsequently calculated a Wilcoxon signed rank test to compare fixation on human and artificial faces in the video category which contained both ROI types. Here, human faces were significantly prioritized (*W* = 1100, *p* < 0.001, *r* = 1.24, see Figure 2A).

### Fixation Latency

In the videos in which a specific ROI was regarded at least once, the real faces were, on average, first gazed at 2.52s after video start in the videos containing only real faces (*SD* = 0.79s, range = 1.35s–5.03s), whereas the artificial faces were first gazed at 1.27s after video start in the videos containing only artificial faces (*SD* = 0.52s, range = 0.65s–3.24s). This difference in latencies was significant (*W* = 1199, *p* < 0.001, *r* = 1.15) but it should be noted that this comparison involved two different sets of video clips. In the videos containing both face types, real faces were, on average, first gazed at 1.91s after video start (*SD* = 0.65s, range = 0.81s–3.61s) and the artificial faces, by contrast, at 2.57s (*SD* = 0.79s, range = 1.37s–4.17s). Fixation latencies were thus significantly reduced for real vs. artificial faces (*W* = 898, *p* < 0.001, *r* = 0.54, see Figure 2B) when both faces were presented simultaneously.

### GLMM Results

We used an incremental approach consisting of nine GLMMs by which we could estimate the individual contributions of each predictor to each model for each video subset. All respective results are summarized in Table 1. Overall, the bootstrapping procedure over 100 iterations showed that both central bias and saliency greatly influenced gaze allocation throughout all video types. However, when the respective ROIs were added as predictors to the models, the explained variance increased significantly as revealed by non-overlapping CIs of the *R*^2^s. A direct comparison between real human and artificial faces in the video subset including both face types additionally showed a higher influence of real human faces (β = 0.289, 95% CI [0.285,0.292]) than artificial faces (β = 0.156, 95% CI [0.153,0.159]) on fixation selection while both predictors contributed significantly to gaze allocation.

**TABLE 1:** Results of incremental generalized linear mixed models (GLMMs) investigating the contribution of individual predictors to gaze patterns.

	**Beta weights of predictors**				
**Video subset**	**Central bias**	**Saliency**	**Human faces**	**Artificial faces**	***R*^2^**
Non-social videos (*n* = 15)	0.410 [0.407, 0.413]	0.518 [0.514, 0.523]			0.296 [0.295, 0.297]
Only human face videos (*n* = 15)	0.209 [0.206, 0.212]	0.548 [0.544, 0.551]			0.210 [0.209, 0.212]
	0.240 [0.237, 0.243]	0.526 [0.522, 0.529]	0.322 [0.317, 0.327]		0.254 [0.253, 0.256]
Only artificial face videos (*n* = 15)	0.198 [0.195, 0.201]	0.440 [0.437, 0.443]			0.180 [0.179, 0.181]
	0.164 [0.161, 0.167]	0.431 [0.428, 0.433]		0.205 [0.202, 0.208]	0.204 [0.203, 0.205]
Human and artificial face videos (*n* = 15)	0.142 [0.139, 0.145]	0.483 [0.479, 0.486]			0.160 [0.159, 0.161]
	0.135 [0.132, 0.138]	0.438 [0.434, 0.441]	0.277 [0.273, 0.280]		0.213 [0.212, 0.215]
	0.145 [0.141, 0.148]	0.456 [0.453, 0.460]		0.131 [0.128, 0.134]	0.172 [0.171, 0.173]
	0.139 [0.135, 0.141]	0.398 [0.394, 0.401]	0.289 [0.285, 0.292]	0.156 [0.153, 0.159]	0.230 [0.229, 0.232]

*Mean beta weights and explained variance (*R*^2^) for models comprising an increasing number of predictors. Models are nested and include predictors in models shown above for the specific set of videos. All values were calculated by bootstrapping 100 sets of not-looked-at grid cells and performing GLMMs for each set. Estimates represent means of weights from each bootstrapping iteration. Values in brackets represent the 2.5th and 97.5th percentile rank as an unbiased estimate of the 95% confidence interval.*

## Discussion

It is generally established that faces elicit an attentional bias toward them. In the current study, we examined whether this attentional bias persists for various face types or whether the presence of real human and artificial faces differentially impacts gaze allocation when viewing videos of complex, naturalistic scenes. While both face types significantly predicted gaze, the relative influence of artificial faces was reduced when real human faces were presented simultaneously. This result was also evident in longer fixation durations on and faster gaze orienting toward real human faces suggesting that real faces are more relevant to observers than artificial ones.

These findings add to pre-existing knowledge on social attention by disentangling the contributions of different face types in naturalistic scenes. Previously, a general strong prioritization of social features (e.g., human heads or bodies) had been described in the literature (Bindemann et al., 2005; Birmingham et al., 2008; Coutrot and Guyader, 2014; End and Gamer, 2017; Flechsenhar and Gamer, 2017; Rösler et al., 2017; Flechsenhar et al., 2018), yet the use of stimulus material varied widely. While many researchers relied on isolated or schematic, artificial faces (e.g., Bindemann et al., 2005, 2008; Theeuwes and Van der Stigchel, 2006), others employed static or dynamic stimuli representing real humans in naturalistic settings (e.g., Birmingham et al., 2008; End and Gamer, 2017). In order to be able to systematically differentiate between artificial and real human faces, we utilized videos containing either only one of the two face types or both human and artificial faces. We were thereby able to see that artificial faces predict gaze when presented exclusively and remain to influence fixations patterns when presented in competition with real human faces. In direct contrast to real human faces, artificial faces yet attracted gaze considerably less as reflected by an enhanced fixation latency, a substantially lower average fixation duration and beta estimate.

These findings are seemingly at odds with a study by Laidlaw et al. (2011) who used mobile eye-tracking to differentiate gaze patterns when participants viewed either a real or a video-taped person in a waiting room scenario. As observers fixated the video-taped person displayed on a PC screen more frequently than the live person in the room, it was hypothesized that humans might reduce eye contact when it could lead to a social interaction. A potential interaction is indeed one key difference between real human and artificial faces, yet in our study none of the two face types truly give room for an interaction. While it is therefore not surprising that we cannot replicate the effects observed by Laidlaw et al. (2011), it would be interesting to investigate the impact of real and artificial faces in live conditions. The general predictive power of artificial faces observed in our study is yet in line with a previous observation that eyes attract gaze even when they are presented on non-human monsters and independent of where they are located on the body (Levy et al., 2013). Similarly, studies investigating human-robot-interactions have shown that people can make use of referential gaze cues elicited by robots (Mutlu et al., 2009) and that this gaze-following already becomes evident during infancy and occurs even for non-humanoid robots (Movellan and Watson, 2002). These findings are further corroborated by a recent study which reported a preference of face-like stimuli in the human fetus, suggesting that our tendency to fixate face-like structures evolves *a priori* (Reid et al., 2017). Face processing is indeed known to occur holistically such that different components of a face are integrated and interpreted together (Maurer et al., 2002; Goffaux and Rossion, 2006; Van Belle et al., 2010). Face inversion disrupts this process leading face recognition accuracies to drastically decline when inverted faces are presented (Yin, 1969). While the vast majority of studies examined face processing in two-dimensional faces, it was recently reported that recognition is improved for 3D vs. 2D faces but not when they are inverted (Eng et al., 2017). This refined recognition is likely due to improved holistic processing when faces are more realistic and depth information is enriched. Similar enhanced holistic processing effects might underlie the increased fixations on real vs. artificial faces in the current study which would potentially result in less pronounced gaze differences when both face types are inversed.

The use of GLMMs further enabled us to investigate the relative contributions of additional predictors on gaze patterns, while allowing for correlations between the individual predictors. Nuthmann and Einhäuser suggested this framework as particularly advantageous for the analysis of gaze during the observation of complex stimuli as their low-level features often tend to be correlated (Nuthmann and Einhäuser, 2015). In all of our models, however, low-level saliency contributed critically to gaze allocation and was even seen to explain eye movements significantly better than faces. It is generally known that both low-level physical saliency and higher-level semantic saliency contribute to attentional selection (Henderson et al., 2007; Einhäuser et al., 2008; Santangelo et al., 2015; Flechsenhar and Gamer, 2017) and a recent review showed that both contribute to the likelihood of an item being remembered (Santangelo, 2015). Considering the higher-level semantic relevance of faces in social scenes, the substantial role of both lower-level physical saliency and the presence of faces in the prediction of gaze provide further support for models which claim that perceptual and semantic saliency drives attentional allocation. Contrary to our observation, Coutrot and Guyader found that faces most prominently influenced eye movements of participants who viewed dynamic conversations, whereas saliency did not crucially account for the recorded gaze (Coutrot and Guyader, 2014). However, while our videos did not contain any relevant auditory information, Coutrot and Guyader solely presented conversations rendering the faces displayed in the scene even more relevant to the understanding of its gist. The observed discrepancy in results once again stresses how many factors need to be taken into account when attempting to investigate the mechanisms underlying gaze allocation in naturalistic scenes. Multisensory approaches as, for instance, employed by Nardo et al. (2014) to study spatial attention, might therefore be helpful in disentangling the various factors influencing the perception of faces in complex naturalistic scenes.

The examination of fixation preferences for real human and artificial faces can also further our understanding of mental disorders in which alterations of gaze behavior are implicated. Although children are overall more susceptible to distractions by physically salient image regions than adults (Cavallina et al., 2018), children with autism spectrum disorder display particularly decreased attention to fellow humans, especially faces (Dawson et al., 1998, 2004) and are less likely to follow gaze than their peers (Leekam et al., 2000). These difficulties do not decline with age (Baron-Cohen et al., 2001; Spezio et al., 2007) and it is generally assumed that the higher-level saliency of social features is reduced for patients with autism-spectrum disorder (Dawson et al., 1998; Klin et al., 2003; Wang et al., 2015). There is some evidence that reduced social attention in autism does not transfer to artificial faces since children with autism spectrum disorder were seen to use regular processing strategies for cartoon faces while processing real faces atypically (Rosset et al., 2008). Additionally, healthy peers performed better in a discrimination task when presented with real vs. cartoon faces, whereas patients with autism spectrum disorder did not exhibit a difference in performance (Rosset et al., 2010). The current study provides additional information on gaze allocation toward real and artificial faces than previously established and thereby offers a more elaborate framework for the examination of gaze alterations in autism.

One potential pitfall of our experimental design is that the majority of artificial faces did not exhibit movement. Although their position within the video could change because of smooth pan shots or slight camera movements, real human faces were more likely to move. However, the GVBS algorithm, which we used to calculate the physical saliency of different image regions, considers movement across frames. In our statistical model, we were therefore able to take a disparate percentage of motion between face types into account and thus assume that the differences in gaze behavior toward artificial vs. real faces cannot be solely explained by motion. Additionally, we need to bear in mind that faces are typically connected to bodies – the extent of which might differ between artificial and real faces in our study. While we attempted to find comparable stimulus material, artificial faces were more frequently presented without being connected to a meaningful bodily extension. Various studies investigating gaze patterns in social scenes (e.g., End and Gamer, 2017, 2019; Flechsenhar and Gamer, 2017) have yet shown that faces attract decisively more fixations than other body parts, and we hence believe that differences in the presence of extremities do not influence our findings gravely.

To conclude, the current study used multiple GLMMs to identify several crucial predictors of gaze allocation when viewing complex dynamic scenes. Saliency and central bias had highest predictive power, while both real human and artificial faces also substantially contributed to the prediction of gaze patterns. Taken together, these findings shed further light on the mechanisms underlying the distribution of social attention and highlight the role both real human and artificial faces play in the visual exploration of our surroundings.

## Data Availability Statement

The raw data supporting the conclusions of this article will be made available by the authors, without undue reservation, to any qualified researcher.

## Ethics Statement

The studies involving human participants were reviewed and approved by the ethics committee of German Psychological Society (DGPs). The participants provided their written informed consent to participate in this study.

## Author Contributions

MR and MG developed the study concept and design. MR performed and supervised the data collection. MR and LR analyzed and interpreted the data under supervision of MG. LR drafted the manuscript. MR and MG provided critical revisions. All authors approved the final version of the manuscript.

## Conflict of Interest

The authors declare that the research was conducted in the absence of any commercial or financial relationships that could be construed as a potential conflict of interest. The handling Editor declared a past co-authorship with one of the authors MG.
