# Supplementary material for: Artificial Faces Predict Gaze Allocation in Complex Dynamic Scenes
Source: Front Psychol. 2019 Dec 18;10:2877. doi: 10.3389/fpsyg.2019.02877 (PMC6930810; doi:10.3389/fpsyg.2019.02877)
Supplement: Supplementary file 1 [file Table_1.DOCX]

Supplementary Material

# Supplementary Table 1.

| **Neutral Videos** | |
| --- | --- |
| 1 | A green canyon with a wild river in the middle and several waterfalls streaming into it. Filmed out of an airplane. |
| 2 | A rollercoaster ride out of the perspective of the cart. You can see the rail track and bald trees along the rollercoaster. |
| 3 | Scene in an automobile factory. You can see a skeleton of a car and many robot arms that are working on it. |
| 4 | Walking along a trail out of a forest to a clearing. There is a river to the left. |
| 5 | Colorful balloons with tags on them are released into the sky. |
| 6 | Two hot-air balloons are floating over a sunny, hilly landscape. |
| 7 | At a train track. You can see a wagon train with machines on it on the left and a normal train on the right, both moving. |
| 8 | A ski-run down a snowy hill out of the perspective of the skier. Entering a forest after a while. |
| 9 | Scene filmed from a boat, looking at the water and the coast with tall buildings and trees. |
| 10 | Lava streaming down a volcanic mountain. There is water at the foot of the mountain and steam rising. |
| 11 | A small boat is struggling in a very rough sea. |
| 12 | A lot of old, rusty vintage cars on a snowy field. |
| 13 | The opening of a watergate filmed from inside the watergate. |
| 14 | The slow passing of a wagon train, filmed from a bridge. There are trees next to the tracks. |
| 15 | A sunflower field, the flowers are moving in the wind. |
| **Real Faces Only Videos** | |
| 16 | Inside an airport hall. There is a woman (**real face**) and a man (**real face**) with luggage on the left, waiting. On the right, an employee (**real face**) is moving luggage carts. In the end, a man (**real face**) walks into the frame from the left. |
| 17 | A field with sheep with a man (**real face**) in reflective clothing standing at the fence, looking at the sheep. The sheep are running towards him. After a while, the man (**real face**) turns around and leaves the frame. |
| 18 | A crane moves a big tree trunk. Two men (**real faces**) in reflective clothing enter the scene and monitor the action and give directions to the person (**real face**) operating the crane. |
| 19 | A big ship enters a harbor with other ships on left and right. A couple of people (**real faces**) are watching. There is something burning in the sand and smoke emerges. |
| 20 | A man (**real face**) in a workshops puts together a wooden object. There are tools laying around. He takes a screwdriver and screws and is explaining what he is doing. |
| 21 | There are three children (**real faces**) playing on a playground. One is on a swing, another tries to get on another swing and the third one is standing in front of them. There are trees in the background and a cyclist (**real face**) passes by. |
| 22 | Indigenous farmers (**real faces**) in the mountains, with a herd of alpacas surrounding them. The camera moves and shows the valley with a river. |
| 23 | Scene in an indigenous village. A woman (**real face**) grills an animal on a fire. A child (**real face**) walks up to her and carries the food in a big palm leaf to a group making music. In the background, you can see people (**real faces**) working with other big plant leaves. |
| 24 | A group of children (**real faces**) is singing in a choir. On the left, there is the choir director (**real face**) singing along and on the right, a man (**real face**) is accompanying the group with a guitar. |
| 25 | Three children (**real faces**) are playing on a trampoline, playfully wrestling for a ball. You can see the neighbourhood in the background. |
| 26 | A scene on a beach, with people (**real faces**) going for a walk or exercising by the water. |
| 27 | A man (**real face**) is cooking food outside of a house in a pan. A woman (**real face**) is standing next to him and a man (**real face**) is sitting in the background, reading a newspaper. There are two motorcycles parked next to the cooking station. |
| 28 | Three young adults (**real faces**) are playing frisbees. One is giving directions. |
| 29 | A scene of a construction site next to a highway. A huge drill creates a hole in the ground and a man (**real face**) in reflective clothes stands next to it and supervises the action. |
| 30 | A girl (**real face**) is slowly riding a horse in a sand square. |
| **Real and Artificial Faces Videos** | |
| 31 | Scene at an amusement park. There is a big locomotive with a face on it (**artificial face**) in the middle with wagons of animal sculptures (**artificial faces**) in the back. There is water coming out of the locomotive and the animal's mouthes. Children and adults (**real faces**) are gathering around it. |
| 32 | The entrance to a Hinduistic temple. There are two sculptures of goddesses (**artificial faces**) next to the entrance. You can see two men inside (**real faces**), who then come out of the temple. |
| 33 | A big human-like stone sculpture (**artificial face**) is moved by people (**real faces**) with two ropes on each side. People (**real faces**) are standing around, either pulling, taking pictures or watching. |
| 34 | A scene from a theatre play. There are two actresses (**real faces**) dressed up as clowns in the middle, acting. On each side of the stage is a clown-statue made out of balloons (**artificial faces**). |
| 35 | Sculptors (**real faces**) are shown making human figurines (**artificial face**) out of clay. |
| 36 | A man and an artist (**real faces**) are posing for pictures in front of a big painting of the man (**artificial face**). |
| 37 | An old man (**real face**) is sitting in a small room stuffed with human-like figures out of clay and straw (**artificial faces**). He is sitting on the floor, making new ones out of straw (**artificial faces**). |
| 38 | A scene inside of a Lego store. There are a lot of customers (**real faces**) looking at products. The room is decorated with big sculptures (**artificial faces**) and pictures of characters from different movies and TV shows (**artificial faces**). |
| 39 | Four men (**real faces**) are standing in front of a wall, on which there are paintings of rugby and baseball players (**artificial faces**). |
| 40 | A scene from a cosplay show. First, a host (**real face**) talks to the audience. Then, people in costumes enter the stage, dressed up as animals or ninjas (**artificial faces**). |
| 41 | A clergyman (**real face**) is sitting in a temple playing an instrument. He is surrounded by several human-like figurines (**artificial faces**). |
| 42 | A scene inside of a subway with a ventriloquist (**real face**), who is performing with his doll (**artificial face**) towards the camera. There are people (**real faces**) in the back watching. |
| 43 | A group of street artists is standing on a scaffold and is busy painting a wall with graffiti letterings and faces (**artificial faces**). |
| 44 | Two men (**real faces**) play a game in which they throw scraps of paper in bowls located on a round, rotating table. In the background of the game are figurines (**artificial faces**) and paintings of people (**artificial faces**). |
| 45 | A man (**real face**) is standing in front of many portrait paintings (**artificial faces**) and giving a presentation to an audience. |
| **Artificial Faces Only Videos** | |
| 46 | A scene from a puppet show in a church. There is one puppet (**artificial face**) shown above a table. In the background are church windows with biblical scenes on them. |
| 47 | A big sculpture (**artificial face**) of the manger scene is shown inside of a church. |
| 48 | Scene walking towards a visitor platform in front of a big human-like stone sculpture (**artificial face**) in a plateau landscape. |
| 49 | Scene filming a wall with graffiti art with letterings and comic characters (**artificial faces**). |
| 50 | Two brazen, realistic sculptures of men (**artificial faces**). |
| 51 | An exhibition on Native Americans. Moving figurines (**artificial faces**) are shown doing different kinds of work in a village. |
| 52 | The entrance of a gothic church with several figurines of clergymen (**artificial faces**). |
| 53 | A big boundary wall with Spanish writing and a painting of a cartoon-like human (**artificial face**) lying on the floor. |
| 54 | A Disney rollercoaster. There is a big poster in the middle with cartoon characters (**artificial faces**) on it. |
| 55 | A scene filmed out of a car. The camera is moving through an entrance gate and passing a buddha statue (**artificial face**) next to the road. There are other cars on the street. |
| 56 | A scene filmed out of a cart of an amusement park ride. On the left and right is a fictional forest landscape with moving animal figurines (**artificial faces**). |
| 57 | A big building with a statue of a meditating man (**artificial face**) in the front. |
| 58 | A big painting of two people (**artificial faces**) and a Mickey Mouse (**artificial face**) standing in a desert catching fire. |
| 59 | A painted wall with many people on them (**artificial face**), riding bikes, sitting by a river, playing sports. |
| 60 | The altar of a temple with many extensively decorated figurines (**artificial faces**). |
